# Brexit: reality bites for health on the island of Ireland

**DOI:** 10.1093/eurpub/ckac178

**Published:** 2022-11-29

**Authors:** Martin McKee, Anthony Staines

**Affiliations:** Faculty of Public Health and Policy, London School of Hygiene & Tropical Medicine, London, UK; School of Nursing, Psychotherapy, and Community Health, Dublin City University, Dublin 9, Ireland

The British government’s decision to leave the European Union has, as was widely predicted, inflicted severe damage on the UK. A series of short-lived governments have limped from crisis to crisis, presiding over progressive economic decline. It was soon clear that the ‘sunlit uplands’ promised by Brexit’s supporters were illusory and Boris Johnson’s claim to have ‘got Brexit done’ was as a fantasy. Yet even if many of those in England and Wales who voted Leave now recognize that they were lied to, the fact is that they were in the (narrow) majority. This was not the case in Scotland or Northern Ireland and it is on the island of Ireland where the damage caused by Brexit is being felt most acutely. Once again, political developments have profound consequences for public health.

In 1998, the Good Friday Agreement (GFA) brought an end (mostly) to the inter-communal violence that had afflicted Northern Ireland for three decades, after the loss of over 3500 lives. It required some compromises but was a huge step forwards for Northern Ireland.

The GFA was facilitated by Ireland and the UK being EU member states. The strict customs procedures in place prior to the single market and customs union had become largely redundant. Border checks remained, but mainly for security purposes, and were a reminder of the division of the island, often targeted by Republican paramilitary organizations. Their removal was part of the confidence building accompanying the GFA.

Brexit created a problem that was obvious to those who understood this situation, even if not, apparently, to many Brexit supporters. Once the UK stated its intention to diverge from EU rules on, for example, product safety, the EU had to establish checks on its external border to prevent inflows of dangerous or sub-standard products, a concern reinforced by the UK’s support for widespread deregulation. The question was where this border should lie. Rebuilding the border checks on the island of Ireland, contrary to the spirit of the GFA, would threaten the associated peace process, which was widely recognized as remaining fragile. Some British commentators fantasized that Ireland might rejoin the UK.

There were two alternatives. The UK could commit to retaining EU standards and customs arrangements, making a physical border unnecessary. This was the choice adopted by Theresa May, while retaining the right to diverge at some future date when the border arrangements would be revisited (the ‘backstop’). However, she was unable to persuade the Conservative Party and resigned. In its place came what became known as the Northern Ireland Protocol. This imposed customs checks between Great Britain (England, Wales and Scotland) and Northern Ireland. This was agreed by Boris Johnson who claimed, entirely falsely, that no actual checks would be needed. Subsequently, it became clear that he never intended to comply with the Protocol he signed.

Meanwhile, problems were emerging in Northern Irish politics. The GFA had created a political system in which representatives of the unionist (identifying with the UK), and nationalist (identifying with Ireland) are required to share power. The largest party appoints a First Minister, with the largest from the other tradition appointing a Deputy. Other posts were allocated across the two communities. The unionists, which had, in various guises, been the largest party since the creation of Northern Ireland were overtaken at the last elections in May 2022, by Sinn Féin, an avowedly nationalist party. As a result, the Democratic Unionist Party, the largest unionist party, has blocked the creation of an executive, leaving Northern Ireland without a government. Their ostensible reason was the unacceptability of the Northern Ireland Protocol and with it, in their view, the weakening of links with Great Britain.

By now, most people in Northern Ireland realize that the Protocol is the only feasible solution to the problems created by leaving the EU. Northern Ireland and London are the only parts of the UK to have achieved post-pandemic economic recovery.^1^ Business in Northern Ireland is overwhelmingly supportive. Yet the British Conservative Party objects on ideological grounds to EU law applying to any part of their country and is now enacting legislation that, it accepts, will breach international law.

This has obvious implications for the UK’s international credibility. It was long seen as a bastion of the rule of law but, no more. However, it also impacts more widely on future EU-UK collaboration. The EU had insisted that the UK agree on three issues before negotiating a Trade and Cooperation Agreement (TCA). These were citizens’ rights, the UK’s financial obligations, and the Irish situation. The UK was now tearing up the foundations on which the TCA had been built. The impasse has prevented the UK participating in EU health research or certain disease control activities.^2^

Meanwhile, in Northern Ireland, the political vacuum is allowing the National Health Service slowly to collapse.^3^ There is no mechanism to implement measures adopted to protect the population elsewhere in the UK from the cost-of-living increase. In these circumstances, there is a real risk of a health crisis. Many less affluent areas, including working class unionist communities, continue to be neglected, with worse health, educational,^4^ and employment outcomes.^5^ There is also a risk that the uncertainty, coupled with unrealistic expectations promulgated by some British politicians, could encourage some extremist groups to return to violence. There have already been concerning signs, such as the burning of effigies of politicians.

In 1921, the unionist politician Edward Carson famously said ‘what a fool I was, I was only a puppet … and so was Ireland, in the political game that was to get the Conservative Party into power’.^6^ Some things seem never to change.

*Conflicts of interest*: M.M. is President of the British Medical Association and Past President of EUPHA but writes in a personal capacity.
